# Deep learning application of vertebral compression fracture detection using mask R-CNN

**DOI:** 10.1038/s41598-024-67017-6

**Published:** 2024-07-15

**Authors:** Seungyoon Paik, Jiwon Park, Jae Young Hong, Sung Won Han

**Affiliations:** 1 School of Industrial and Management Engineering, Korea University, Anam-ro 145, Seongbuk-gu, Seoul, 02841 South Korea; 2 Department of Orthopaedic Surgery, Korea University Ansan Hospital, 123, Jeokgeum-ro, Danwon-gu, Ansan, Gyeonggi-do South Korea

**Keywords:** Image processing, Machine learning

## Abstract

Vertebral compression fractures (VCFs) of the thoracolumbar spine are commonly caused by osteoporosis or result from traumatic events. Early diagnosis of vertebral compression fractures can prevent further damage to patients. When assessing these fractures, plain radiographs are used as the primary diagnostic modality. In this study, we developed a deep learning based fracture detection model that could be used as a tool for primary care in the orthopedic department. We constructed a VCF dataset using 487 lateral radiographs, which included 598 fractures in the L1-T11 vertebra. For detecting VCFs, Mask R-CNN model was trained and optimized, and was compared to three other popular models on instance segmentation, Cascade Mask R-CNN, YOLOACT, and YOLOv5. With Mask R-CNN we achieved highest mean average precision score of 0.58, and were able to locate each fracture pixel-wise. In addition, the model showed high overall sensitivity, specificity, and accuracy, indicating that it detected fractures accurately and without misdiagnosis. Our model can be a potential tool for detecting VCFs from a simple radiograph and assisting doctors in making appropriate decisions in initial diagnosis.

## Introduction

Vertebral compression fractures (VCFs) are breaks or cracks in the vertebrae, which can cause the spine to weaken or collapse. VCFs affect approximately 1 to 1.5 million people annually in the United States^1^. Although some VCFs are caused by trauma or tumors, they are more common in the elderly and women with osteoporosis. Most VCFs occur in the thoracic and lumbar vertebrae, or at the thoracolumbar junction. In the diagnosis of VCFs, plain radiographs are the initial diagnostic modality. When neurological disorder is suspected, other more complex modalities such as computed tomography (CT), magnetic resonance imaging (MRI) are ordered. Identifying fractures in bone images is a time-consuming and labor-intensive process, that requires manual inspection by a highly trained radiologist or an orthopedic^2^. Inexperience of the clinician or fatigue caused by excessive workloads of physicians can lead to an inaccurate diagnosis, which can be fatal to patients.

Deep learning (DL) algorithms, particularly convolutional neural networks (CNN), have become a powerful method in medical imaging diagnosis^3,4^. Because they are designed to learn spatial hierarchies of features through convolution layers, they are widely used in computer vision tasks such as image classification, object detection, and segmentation. Many studies dealt with identifying bone fractures of various areas of the body using medical images^5–7^. Recently, there have been numerous studies on the use of CNN-based algorithms to assist spinal disease diagnosis including vertebral fractures. Some studies proposed segmentation models for the vertebrae^8–11^. These studies utilized detection and segmentation models, and they approached VCF diagnosis as a two-step process of segmenting every vertebra and the evaluating each of them. Other studies applied CNN-based models for classification of radiograph for diagnostic purposes^12–15^.

However, there have been very few studies dealing with the detection of vertebral fractures on X-rays due to several reasons. It is difficult to acquire a sufficient amount of radiographs of the spine for a specific fracture compared to other fractures of the body, because radiographs are not used for a final diagnosis. Moreover, the labeling process for each fracture on the radiograph is very labor-intensive and challenging, because even experts should match each radiograph with CT or MRI results to find the ground truth. Existing studies regarding the diagnosis of vertebral fractures with DL algorithms are mostly focused on the classification of the medical image or the segmentation of each vertebra. It can be observed that most of the existing works have focused on the classification of each medical image, or considered a two-step process of evaluating fractures after segmenting every spine. In this study, (1) we constructed a high-quality dataset of VCFs of L1-T11 vertebra on lateral spinal X-rays, which were annotated based on the MRI results; (2) subsequently, we proposed a pipeline of training and optimizing a highly accurate Mask R-CNN model to directly locate and classify the fracture and compared it with other popular CNN-based models; (3) and finally, we showed the feasibility of developing a generalized deep learning based diagnosis tool and widened the possibility of real-world use of the model to assist doctors in detection VCFs.

## Materials and methods

### Data source and preprocessing

The dataset used in this study was obtained as lateral thoracolumbar radiographs of patients from Ansan Hospital, the University of Korea. The collected dataset contained 487 radiographs with fractures, and 141 normal radiographs. Only X-rays confirmed as compression fractures based on MRI results were collected and labeled. The X-ray was de-identified before being used, so that each patient’s personal information was removed according to the ethical guidelines. Overall, 598 segmentation masks of marked fractures were extracted from 487 lateral thoracolumbar X-rays and used to train and test each model.

**Figure 1:**
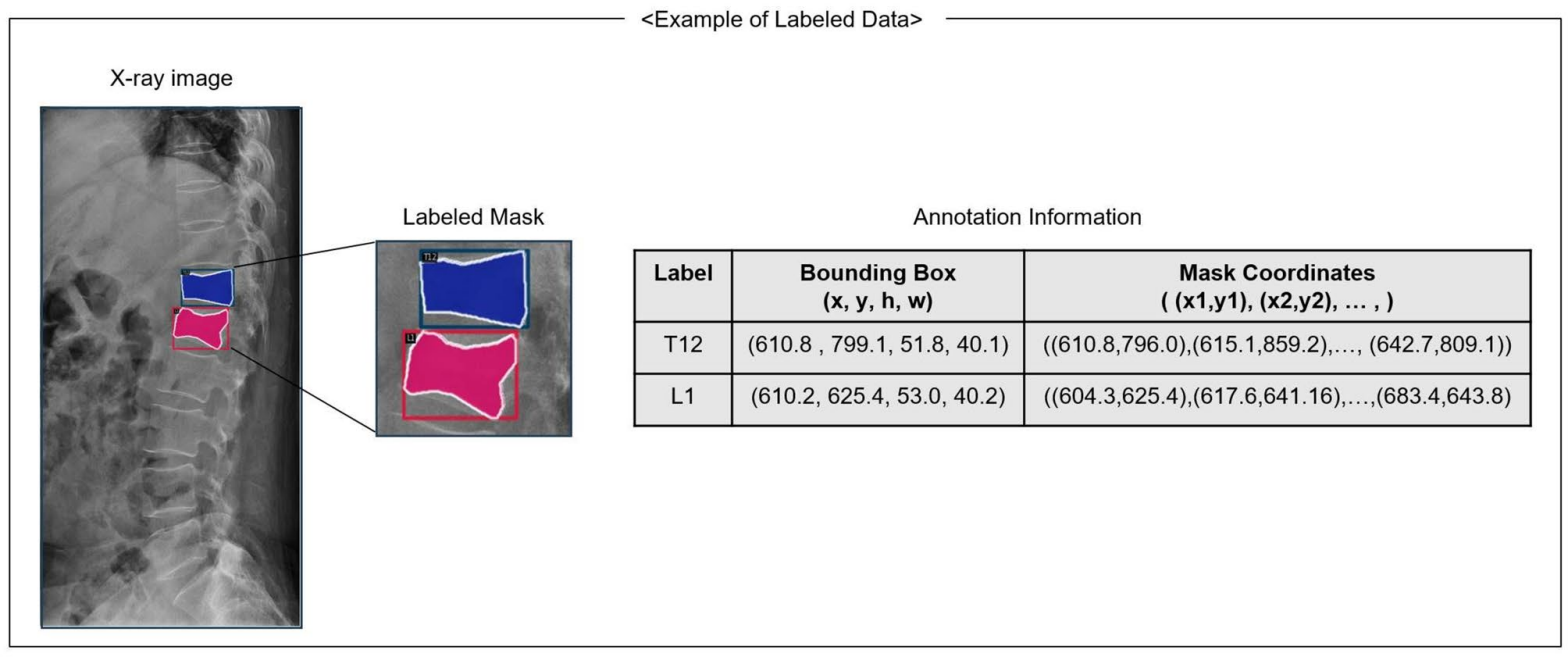
Example of labeled data. Each fracture is labeled with a polygon on the thoracolumbar radiograph based on the MRI results. Each polygon mask contains the x and y coordinates of the polygon mask surrounding it. Each bounding box consists of upper left x, y coordinates, width, and height. The entire labeling process was conducted by two trained orthopedic experts.

A total of six MRI-based class labels were defined and locations were marked during data preprocessing : L1 , L2 , L3 , L4 , T11, T12 fractures. Two orthopedic experts labelled the location and the type of vertebra, using an open source labeling software ‘labelme’, version 5.0.2 (https://github.com/labelmeai/labelme)^16^. Each polygon mask included fracture information on fractures in the six classes (L1-T11), and coordinates of identified fractures at each point of the polygon. Figure 1 shows an example of labeled data used in training. Multiple VCFs were identified in approximately 20% of the patients, and were also labeled as separate polygons.

### Study settings

In this study, approximately 70% (346 radiographs) of the dataset were used to train the neural network, and approximately 15% each were allocated to validation (71 radiographs) and test data (70 radiographs). Train, validation, test data were split in a stratified manner to consider classwise distribution. Radiographs with no fractures were used only in the test phase. We used stochastic gradient descent considering momentum as the optimization method. The learning rate was set to decrease with a weight decay of 0.0001 and a momentum of 0.9. We used transfer learning^17^ to enhance model performance. Each model was trained starting from the pretrained weights of the COCO instance segmentation dataset^18^. Augmentation of horizontal flip and random rotation of 10 degrees were applied. The summary of our VCF dataset is listed in Table 1.

**Table 1:** Summary of VCF dataset.

VCF findings	Total	Train	Valid	Test	# of fractures
T11	18	10	4	4	30
T12	111	79	16	16	152
L1	138	100	19	19	188
L2	76	57	10	9	113
L3	43	31	6	6	73
L4	18	10	4	4	41
Multi fractures	83	59	12	12	–
No fracture	141	–	–	141	0
Sum	628	346	71	241	598

### Mask R-CNN

**Figure 2:**
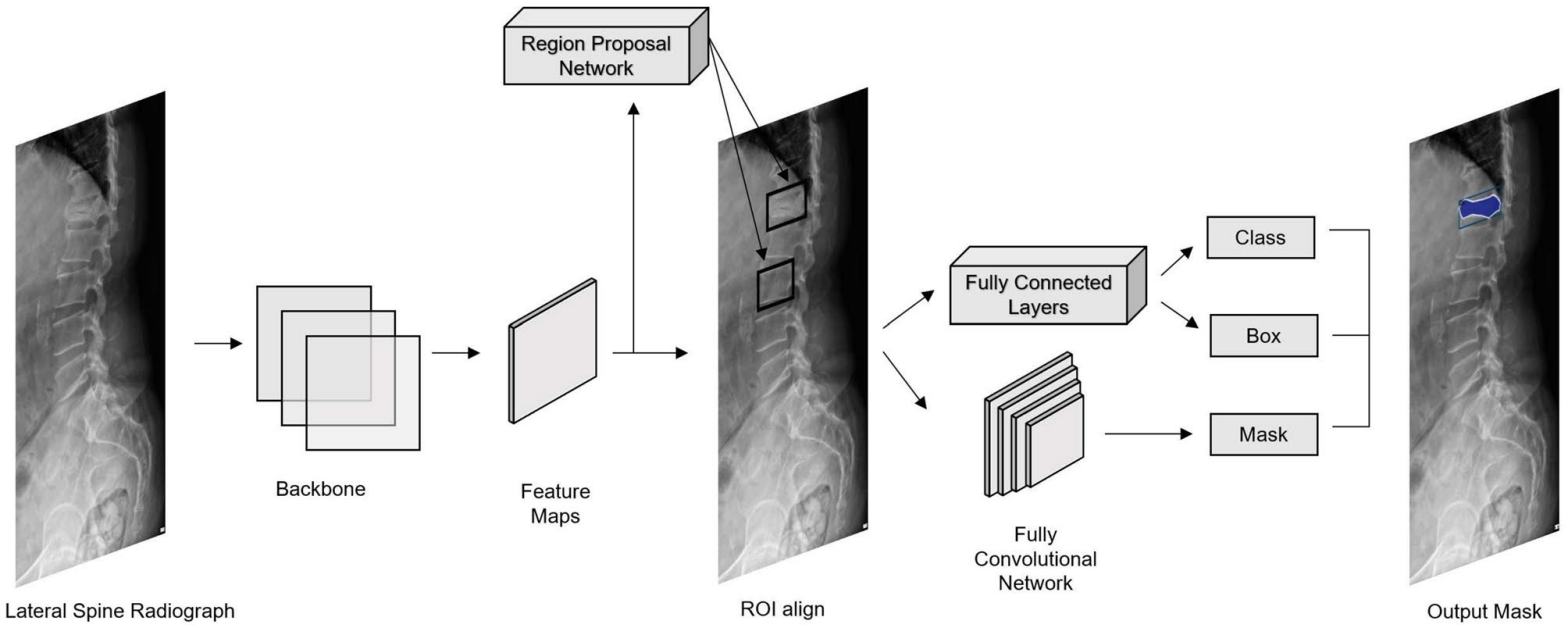
Mask R-CNN model architecture.

Mask R-CNN is an instance segmentation model based on the Faster R-CNN model^19^. Mask R-CNN^20^ introduced the segmentation branch, which is composed of four convolutions, one deconvolution, and one convolution to process instance segmentation. Moreover, ROI Align was introduced to fix the information loss of ROI pooling due to the misalignment of feature maps and ROIs (Region of Interest), and significantly improved the segmentation accuracy. The backbone of Mask R-CNN is ResNet^21^ and Feature Pyramid Networks (FPN)^22^. The backbone used residual learning to precisely extract object features, and a feature pyramid to fuse multi-scale features to construct high-quality feature maps. Subsequently, ROIs were extracted from the feature maps using region proposal networks (region proposal networks). The ROIs were then aligned and pooled by ROI Align. After the pooling layer, the model predicted segmentation masks using fully convolution networks. The structure of Mask R-CNN is shown in Fig. 2. Mask R-CNN has several applications in instance segmentation. Mask R-CNN incorporated the structure of previous RCNN models and improved them to make a faster, more accurate, and more effective instance segmentation model.

#### Backbone network

**Figure 3:**
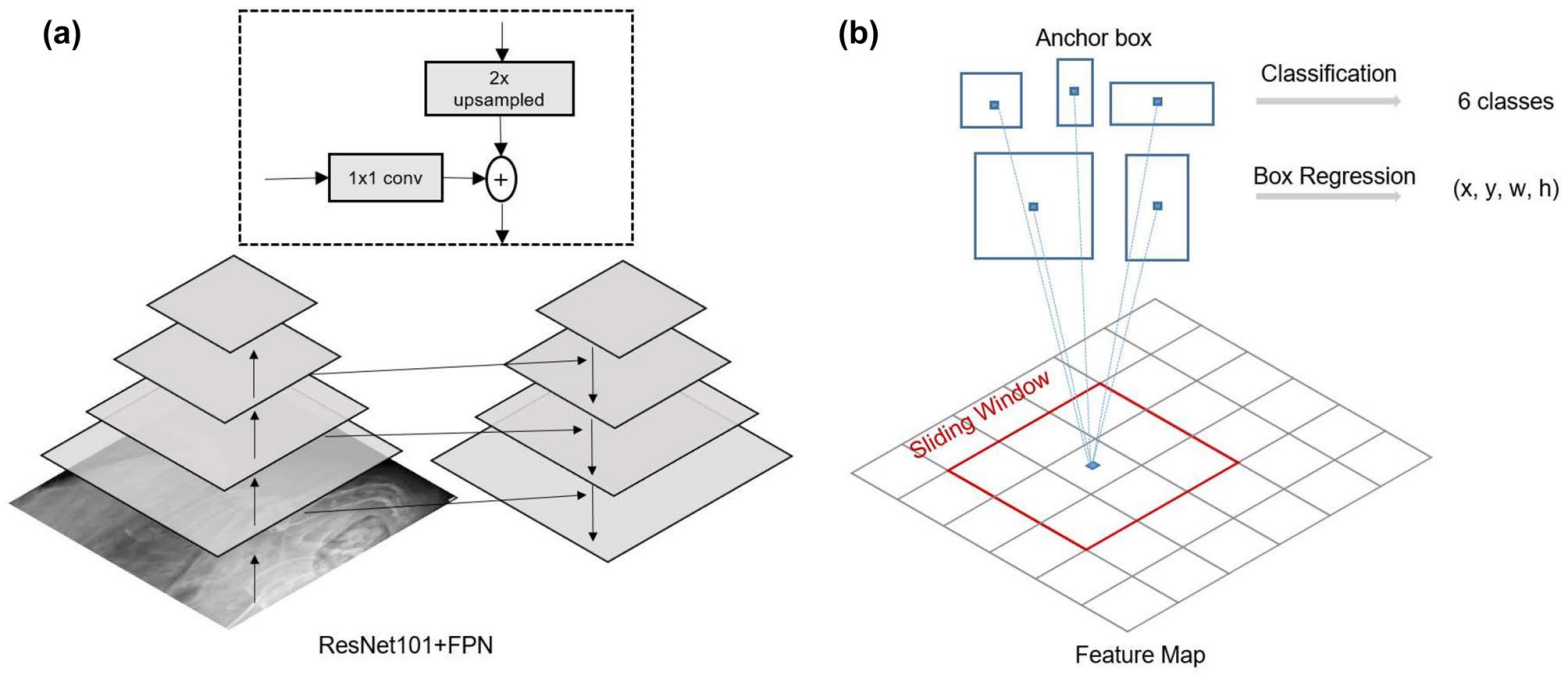
Backbone network and region proposal network. (**a**) Backbone network is shown. Feature maps from ResNet are upsampled and resized with 1 x 1 convolution to be concatenated with different scaled feature maps. (**b**) The region proposal network generates candidate regions for objects by sliding-window, referred to as anchor box on feature maps. Each anchor box performs both classification and bounding box adjustments.

The backbone network was used to extract features from the input radiograph. We implemented ResNet101 with FPN as the backbone network to extract reliable feature maps. In the bottom-up pathway, ResNet extracts low-level features such as corners and edges of the object, while deeper layers extract high-level features such as texture and color. Then in the top-down pathway, FPN was used to concatenate feature maps of different scales to better represent objects. The feature maps were used in the RPN and ROI Align to generate candidate region proposals for detection. The structure of the backbone network is shown in Fig. 3.

#### Region proposal network and ROI align

The RPN generates ROIs using the feature map inputs from the backbone network. A 3 x 3 convolutional layer was used to scan the image using a sliding window to generate anchors for different scaled bounding boxes. Binary classification was performed to determine whether each anchor contained the object or represented a background. The structure of the RPN is shown in Fig. 3. The bounding box regression generated samples and calculated the intersection over union (IoU) value. If the sample had IoU higher than 0.7, it was defined as a positive sample, otherwise a negative sample. Non-maximum suppression (NMS) was applied to keep regions with the highest confidence score. The feature maps from the backbone network and ROIs from RPN were passed to ROI Align for pooling. ROI Align was performed next stage to obtain fixed size feature vectors and feature maps. ROI Align is a method proposed to avoid misalignment issues identified in the ROI pooling layer, which rounds the locations of the ROIs on the feature map. A bilinear interpolation operation was performed on the sample points in each grid cell before pooling.

#### Mask prediction

The feature vector output from the previous stage was used to calculate the class probabilities of each ROIs for classification, and to train bounding box regressors to refine the location and size of the bounding box to accurately include each object. The mask branch predicted binary masks for each ROI classwise using fully convolutional network (FCN).

### Evaluation metrics

True and False positives were defined by the value of the IoU. IoU was calculated by dividing the overlap between the predicted area and the ground truth area by the union of these. If the IoU of the predicted and actual regions exceeded a certain threshold, the detector’s prediction was determined to be correct, and it was defined as True Positive (TP). On the contrary, if the IoU value was less than the threshold, the result was defined as False Positive (FP). When the detector failed to predict any fracture, it was defined as False Negative (FN). Specificity was calculated using the dataset containing no fractures. We defined True Negative (TN) when the detector did not predict any fractures from normal radiographs, and false detection as False Positive (FP). We calculated sensitivity, specificity, accuracy, and F1-score with the defined confusion matrix.Sensitivity is calculated with Eq. (1), specificity with Eq. (2), accuracy with Eq. (3), and F1-score with Eq. (4)

The cumulative value was determined by listing the detected regions in order of confidence score. As the regions were listed, we calculated a precision-recall curve with the accumulated values and computed the AP from the area below. Mean average precision (mAP) was calculated as the average AP score of each class and evaluated as an overall evaluation metric among each DL models. AP scores were computed with Eq. (5).

1 $$\begin{aligned} Sensitivity&= \frac{TP}{TP + FN} \end{aligned}$$

2 $$\begin{aligned} Specificity&= \frac{TN}{FP + TN} \end{aligned}$$

3 $$\begin{aligned} Accuracy&= \frac{TP + TN}{TP + FP + TN + FN} \end{aligned}$$

4 $$\begin{aligned} F1-score&= 2 * \frac{Precision \times Recall}{Precision + Recall} \end{aligned}$$

5 $$\begin{aligned} AP&= \frac{1}{6}\sum _{confidence}Precision(Recall) \end{aligned}$$

### Ethical approval and consent to participants

This study was conducted according to the Helsinki declaration. This study was approved by the Institutional Review Boards of Korea University Ansan Hospital, and was conducted in accordance with the approved study protocol (IRB No. 2022AS0198). Due to the retrospective nature of this study, informed consent was waived by Korea University Ansan Hospital Institutional Review Board and Ethical Committee.

## Results

We compared the outcomes of Mask R-CNN models with Cascade Mask R-CNN^23^, YOLOACT^24^, and YOLOv5^25^ models in terms of diagnostic accuracy and detection performance. The diagnostic performance of models were compared in Table 2. Mask R-CNN model and YOLOv5-m showed the highest sensitivity of 79.8% and 78.7%, among the four models. Models with high sensitivity did not miss identifying patients with fractures. The Mask R-CNN model and the Cascade Mask R-CNN model had the highest specificity of 89.4% and 90.0%, respectively. However, considering that the Cascade R-CNN model had the lowest sensitivity, this model had a strong tendency to predict that there was no fracture. This indicates that the Mask R-CNN model was able to learn features of each vertebra without fracture through negative samples, which were determined as background from RPN. Mask R-CNN and YOLOv5-m model showed higher F1-score of 82.6 and 83.5 than other models, which meant they more likely classified X-ray images that contained fractures. The Mask R-CNN showed the highest accuracy of 85.7% and provided the best diagnosis among all models.

**Table 2:** Comparison of detection performances.

Model	TP	FP	TN	FN	Sensitivity (%)	Specificity (%)	Accuracy (%)	F1-score
Mask R-CNN	71	15	126	18	79.8	89.4	85.7	82.6
Cascade R-CNN	59	14	127	30	66.3	90.0	80.8	70.7
YOLOACT	60	32	109	29	67.4	77.3	73.4	70.6
YOLOv5-l	64	31	110	25	71.9	78.0	75.7	79.5
YOLOv5-m	70	26	115	19	78.7	81.6	80.4	83.5

The detection performance of models were compared in Table 3. The AP score was computed when the IoU threshold was set to 0.5, and mAP was calculated as the classwise average AP scores. The Mask R-CNN model and the YOLOv5-x model showed the highest mAP of 0.58 compared to others. However, there were differences in detection performance by category, as there was a class imbalance in the collected dataset. For T12, L1, and L2 fractures, of which more than 100 fractures were collected, all models except Cascade Mask R-CNN achieved a mAP higher than 0.7 and showed great detection performance. In contrast, AP scores for T11 and L4 fractures were low for every model, for which a relatively small amount of data was collected. Mask R-CNN model achieved AP score of 0.78, 0.80, and 0.79 for each fractures. The YOLOv5 model with a higher number of model parameters and size showed a higher AP score, as the YOLOv5-x and YOLOv5-l models showed better performance than the YOLOv5-s and YOLOv5-m models.

**Table 3:** Comparison of segmentation AP.

Models/fractures	L1	L2	L3	L4	T11	T12	Overall mAP
Mask R-CNN	0.80	0.79	0.66	0.32	0.10	0.78	0.58
Cascade mask R-CNN	0.63	0.58	0.33	0.24	0.07	0.52	0.40
YOLOACT	0.70	0.66	0.67	0.30	0.22	0.67	0.54
YOLOv5-s	0.72	0.78	0.72	0.24	0.09	0.68	0.54
YOLOv5-m	0.73	0.77	0.67	0.25	0.02	0.68	0.52
YOLOv5-l	0.78	0.82	0.69	0.13	0.07	0.75	0.54
YOLOv5-x	0.78	0.83	0.78	0.54	0.12	0.72	0.58

**Figure 4:**
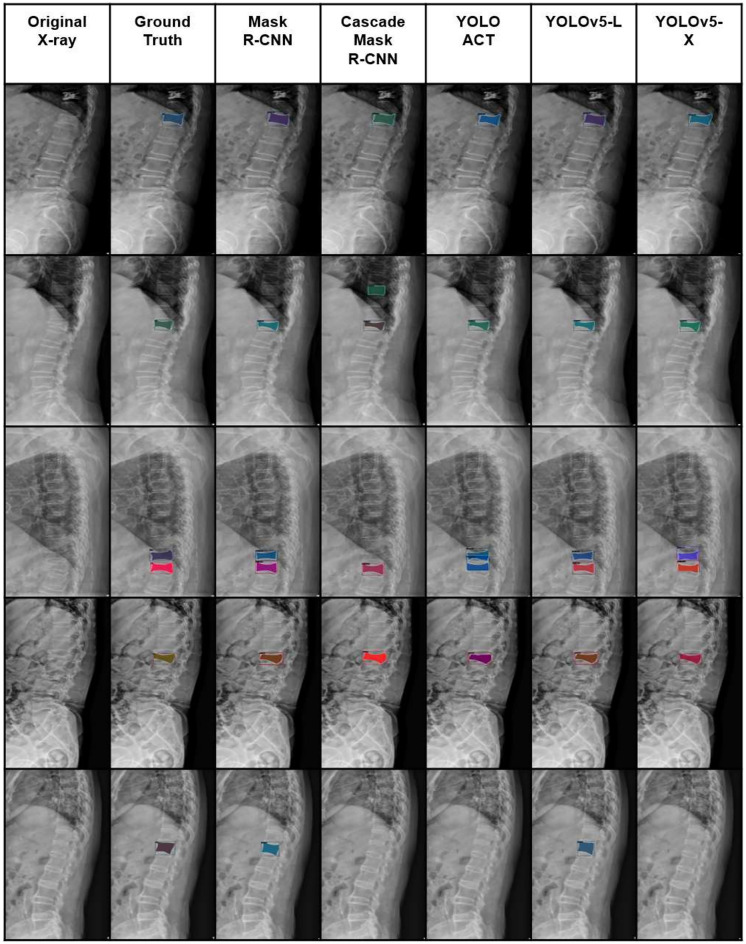
Examples of prediction results of each model. From left to right, actual spine lateral radiograph, ground-truth (expert-labeled fracture masks), and prediction from each model are shown.

Model prediction is shown in Fig. 4. Compared with the ground truth, all the models predicted fractures close to the actual area. Compared to other models, the Mask R-CNN model did not miss any fractures and performed better. Multiple fractures were detected as well. YOLOACT and YOLOv5 models predicted more fracture area as multiple fractures even when only one fracture existed.

## Discussion

Many people are diagnosed with VCFs every day. The plain radiograph of the spinal region is the initial diagnostic method because they are quick and require low-cost compared with other diagnostic modalities^2^. Research on applying DL algorithms to the field of medical image diagnostics is actively being studied and showing good performance^4^. These works can lead to assisting medical experts by reducing the time required in diagnosis and increase accuracy, ultimately improving patient care. However, there is not much research on detecting VCFs by applying DL algorithms for several reasons. Despite the advantages radiograph have in diagnosis of VCFs, it is difficult to construct a large dataset of radiographs of VCFs, because radiographs are not used for a final diagnosis. Also, labeling ground truth on each radiograph is very hard and labor-intensive because MRI findings must be referenced. Thus, previous studies have classified vertebral radiographs or performed segmentation of each vertebra and classified each bone by a two-step process.

From this perspective, this study shows interesting results. We created a VCF dataset labeled based on the MRI findings and trained segmentation deep learning models to diagnose and segment each fracture area. In terms of diagnose, Mask R-CNN and YOLOv5-m model achieved sensitivity of nearly 80%, and F1-score of over 80% (Table 2). For classes which were easier to collect such as L1, L2, T12 most segmentation models achieved an AP score of over 0.7, indicating that they were able to accurately locate fracture regions pixel-wise (Table 3). The results showed that these models were able to successfully distinguish VCFs of patients, highlighting the potential to assist physicians in real-life (Fig. 4). However, there were some limitations in our study. First, the overall performance of the models was not sufficiently high. The most explainable reason for this is the small amount of data on certain classes such as T11, L3, L4 fractures. Also, we only targeted fractures of the thoracolumbar area from L1 to T12, which makes partial diagnose of VCFs. Nevertheless, our study showed and proposed a pipeline for directly locating and classifying fracture by utilizing deep learning segmentation model, Mask R-CNN. In the future, we plan to collect more lateral radiographs and train a model that targets all areas of vertebral body.

The aim of the study was to develop a model that could efficiently detect vertebral compression fractures of the thoracolumbar region in lateral radiographs. We constructed an MRI-based labeled VCF dataset. Subsequently, we trained and optimized DL based instance segmentation model Mask R-CNN. We compared detection and diagnosis performance results with those of other popular segmentation models, and showed that Mask R-CNN was the most appropriate model for detecting VCFs. Deep learning based VCF detection model can reduce time and cost spent on inspection of radiographs manually. If this model is used to assist doctors with the initial diagnosis of VCFs, faster diagnosis is possible and patients’ treatment periods can be shortened.

## Data Availability

Researchers may send reasonable requests for access to the datasets used in this study to the corresponding author.

## References

[1] Alexandru D, So W (2012). Evaluation and management of vertebral compression fractures. Perm. J..

[2] Joshi D, Singh TP (2020). A survey of fracture detection techniques in bone x-ray images. Artif. Intell. Rev..

[3] Shen D, Wu G, Suk H-I (2017). Deep learning in medical image analysis. Annu. Rev. Biomed. Eng..

[4] Litjens G (2017). A survey on deep learning in medical image analysis. Med. Image Anal..

[5] Wang Y-N (2024). A deep-learning model for diagnosing fresh vertebral fractures on magnetic resonance images. World Neurosurg..

[6] Hardalaç F (2022). Fracture detection in wrist x-ray images using deep learning-based object detection models. Sensors.

[7] Uysal F, Hardalaç F, Peker O, Tolunay T, Tokgöz N (2021). Classification of shoulder x-ray images with deep learning ensemble models. Appl. Sci..

[8] Cheng L-W (2023). Automated detection of vertebral fractures from x-ray images: A novel machine learning model and survey of the field. Neurocomputing.

[9] Konya S (2021). Convolutional neural network-based automated segmentation and labeling of the lumbar spine x-ray. J. Craniovertebr. Junct. Spine.

[10] Kim KC, Cho HC, Jang TJ, Choi JM, Seo JK (2021). Automatic detection and segmentation of lumbar vertebrae from x-ray images for compression fracture evaluation. Comput. Methods Progr. Biomed..

[11] Seo JW (2021). A deep learning algorithm for automated measurement of vertebral body compression from x-ray images. Sci. Rep..

[12] Yeh L-R (2022). A deep learning-based method for the diagnosis of vertebral fractures on spine MRI: Retrospective training and validation of resnet. Eur. Spine J..

[13] Hong N (2023). Deep-learning-based detection of vertebral fracture and osteoporosis using lateral spine x-ray radiography. J. Bone Miner. Res..

[14] Chen W (2022). A deep-learning model for identifying fresh vertebral compression fractures on digital radiography. Eur. Radiol..

[15] Murata K (2020). Artificial intelligence for the detection of vertebral fractures on plain spinal radiography. Sci. Rep..

[16] Russell BC, Torralba A, Murphy KP, Freeman WT (2008). Labelme: A database and web-based tool for image annotation. Int. J. Comput. Vis..

[17] Pan SJ, Yang Q (2009). A survey on transfer learning. IEEE Trans. Knowl. Data Eng..

[18] Lin, T. *et al.* Microsoft COCO: common objects in context. *CoRR* https://arXiv.org/abs/1405.0312 (2014).

[19] Ren S, He K, Girshick R, Sun J (2015). Faster r-cnn: Towards real-time object detection with region proposal networks. Adv. Neural Inf. Process. Syst..

[20] He, K., Gkioxari, G., Dollár, P. & Girshick, R. Mask r-cnn. In *Proc. of the IEEE International Conference on Computer Vision*, 2961–2969 (2017).

[21] He, K., Zhang, X., Ren, S. & Sun, J. Deep residual learning for image recognition. In *Proc. of the IEEE Conference on Computer Vision and Pattern Recognition*, 770–778 (2016).

[22] Lin, T.-Y. *et al.* Feature pyramid networks for object detection. In *Proc. of the IEEE Conference on Computer Vision and Pattern Recognition*, 2117–2125 (2017).

[23] Cai, Z. & Vasconcelos, N. Cascade r-cnn: Delving into high quality object detection. In *Proc. of the IEEE Conference on Computer Vision and Pattern Recognition*, 6154–6162 (2018).

[24] Bolya, D., Zhou, C., Xiao, F. & Lee, Y.J. Yolact: Real-time instance segmentation. In *Proc. of the IEEE/CVF International Conference on Computer Vision*, 9157–9166 (2019).

[25] Jocher, G. Ultralytics yolov5, 10.5281/zenodo.3908559 (2020).

